# Parent to Child Intergenerational Transmission of Direct and Indirect Weight and Shape Communication

**DOI:** 10.1007/s10826-025-03078-z

**Published:** 2025-08-14

**Authors:** Emily Ferrer, Shannon Marhan, Leora Haller, Shannon M. O’Connor

**Affiliations:** 1 Department of Psychology, Montclair State University, Montclair, NJ USA; 2 Department of Child and Adolescent Psychiatry, Children’s Hospital of Philadelphia, Philadelphia, PA USA; 3 Department of Psychology, University of Toledo, Toledo, OH USA

**Keywords:** Body talk, Intergenerational, Parents, Parent modeling, Weight conversations

## Abstract

Parental communication about body weight and shape is associated with offspring’s eating behaviors and body image. However, predictors of parental weight/shape communication are less known. The present study explored whether direct (i.e., comments to the child about their weight and encouragement to diet) and indirect (i.e., modeling of the importance of body weight/shape via parent’s own dieting and comments about their own weight) communication about weight, shape, and eating from parents in childhood predicted women’s direct and indirect communication about weight/shape to their own child in adulthood. Participants were 634 adult mothers who provided retrospective reports of their parents’ direct and indirect weight/shape-related communication during childhood. Participants then self-reported their own current direct and indirect weight/shape communication towards their own child. Multiple linear regression explored whether childhood direct and indirect communication predicts maternal weight/shape communication towards participants’ own child in adulthood. Childhood direct weight/shape communication was a salient predictor of both current direct and indirect weight/shape communication. Childhood indirect weight/shape communication did not predict current direct communication when modeled with childhood direct communication, however, it was predictive of current indirect communication. Findings may highlight a need for parental psychoeducation on the lasting influence of familial weight/shape communication.

## Highlights

- Women who experienced parental weight-related comments in childhood is associated with making similar weight-based comments as mothers.
- Additionally, women experiencing parental weight commentary in childhood predicts dieting and parental body talk in adulthood.
- Observing parental dieting/body talk in childhood predicts similar behavior in adulthood for mothers.
- Parental weight/shape talk may be a modifiable risk factor to reduce disordered eating for future generations and warrants further research.

Approximately one in three parents discuss body weight with their child (Berge et al., 2014). Parent-child communication about the importance of body weight and shape is associated with offspring’s eating behaviors and attitudes towards their own bodies (Abraczinskas et al., 2012; Arroyo et al., 2017; Berge et al., 2018; Berge et al., 2024; Claydon et al., 2019; Dahill et al., 2021; Martini et al., 2020; Norton et al., 2022; Rodgers et al., 2009; Rodgers & Chabrol, 2009; Savage et al., 2007; Stice, 1998). Parents may communicate messages about weight/shape to their children directly (e.g., verbal comments to the child about their weight or physical appearance, encouragement to diet or exercise to lose weight) and indirectly (e.g., parents modeling the importance of body weight/shape through their own dieting habits, commenting on their own or other’s weight/shape) (Abraczinskas et al., 2012; Vincent & McCabe, 2000; Lydecker et al., 2018).

Both direct and indirect parental communication about weight and shape has been associated with several poor physical and mental health outcomes in children (Nickelson et al., 2012), such as depression, anxiety (Gillison et al., 2016) and, most commonly, disordered eating and dysfunctional weight loss practices (e.g., food restriction, purging, food obsession, dieting, and binge-eating) (Arroyo et al., 2017; Berge et al., 2018; Claydon et al., 2019; Gillison et al., 2016; Lydecker et al., 2018; Rodgers et al., 2009; Tan et al., 2016). Notably, while there has been research to support that broader health-related conversations with children are associated with positive outcomes (Yourell et al., 2021), conversations that steer into specific weight/shape topics are associated with increased body dissatisfaction in children (Ricciardelli et al., 2003) and into later adulthood (Kluck, 2010; Wansink et al., 2017).

Disordered eating and dysfunctional weight loss practices in children may lead to clinical eating disorders (Herle et al., 2019; Murray et al., 2022; Kotler et al., 2001), such as anorexia nervosa, bulimia nervosa, or binge eating disorder—the former being the second deadliest mental disorder behind substance use disorder (Chesney et al., 2014). Indeed, medical complications associated with clinical eating disorders include many life-threatening conditions involving almost every organ system in the human body, such as cardiovascular (e.g. bradycardia, hypotension, heart failure), gastrointestinal (e.g. gastroparesis, constipation, acid reflux or esophageal damage due to purging), endocrine and metabolic (e.g. hypoglycemia, amenorrhea, osteopenia/osteoporosis, and life threatening electrolyte imbalances), neurological (e.g. cognitive impairment), hematologic (e.g. anemia, low white blood cell and platelet counts), dermatological (e.g. brittle and dry skin and nails), and psychological (e.g. depression, anxiety, suicidal ideation) complications (Downey et al., 2024; Peebles & Sieke, 2019).

Research has tried to grasp an understanding of what factors may predict parental engagement in weight/shape talk, such as parental weight bias (Pudney et al., 2019; Pudney et al., 2023), parent’s own current weight status (Berge et al., 2013) and child’s BMI (Berge et al., 2014; Lydecker et al., 2018; Puhl et al., 2022). Indeed, Pudney et al., (2019) found that internalized weight stigma in parents lead to increased comments on their own and other’s weights to their child. Berge et al. (2013) found that parents in larger bodies tend to have more frequent discussions about weight and the importance of weight loss with their children. Other studies have found a positive association between parental weight and shape communication and child BMI (Berge et al., 2014; Lydecker et al., 2018; Puhl et al., 2022). Research uncovering factors contributing to parent weight/shape talk is growing, however, research exploring factors driving different types of parent communication styles (i.e., direct vs. indirect communication) is still limited.

Importantly, parents’ own childhood experience of communication about weight/shape from their parents may shape their weight/shape communication style in adulthood. Indeed, parents may learn how to communicate about weight/shape from their own experiences of how weight/shape was discussed or modeled within their childhood home. For instance, individuals who received encouragement to diet or commentary about their weight as children may be more likely to comment about their own child’s weight. Similarly, individuals who observed their parents modeling the importance of weight and shape through dieting or commentary about their bodies may be more likely to model these behaviors within their home as adults.

To our knowledge, only two studies have explored this possibility. Berge et al. (2018) used a longitudinal approach with a diverse population based on sex, socioeconomic status, race and ethnicity (*n* = 556; 64.6% female). This study was part of Project EAT which included a four-wave design between 1998–2016 starting when participants were in their teens (mean age of 15.3 ± 1.4 years) to adulthood (mean age of 31.4 ± 1.5). This study demonstrated that individuals who reported parental encouragement to diet in adolescence were more likely to talk to their child about his/her weight and encourage their child to diet as adults (Berge et al., 2018). Additionally, compared to individuals who did not receive parental encouragement to diet, adults who experienced encouragement to diet in adolescence were also more likely to complain about their own weight in the presence of family members and report that other family members within their household talk about their own weight. Experiencing diet talk in adolescence was not significantly associated with family members teasing one another about their weight in adulthood (Berge et al., 2018). Notably, these findings reflect models that were adjusted for sex, race and/or ethnicity, age, family SES, and weight status in adolescence. Berge et al. (2024) reinforced their previous findings in another longitudinal study that collected data from parents of children aged 5–9 (*n* = 1307) across two time points, 18 months apart. Similar to their earlier findings (Berge et al., 2018), parents who were encouraged to diet in their childhood engaged in the same type of encouragement with their own children, and further, this parent engagement in weight talk was associated with higher weight status, poor psychosocial outcomes (e.g., conduct problems, peer relationships problems), and more restrictive feeding practices 18 months later. Taken together these findings provide some evidence for an intergenerational transmission of weight/shape communication, such that experiencing encouragement to diet in adolescence may predict an individual’s future weight-based communication style with their children.

The present study sought to extend prior findings using a large independent sample of mothers (*n* = 634). While Berge et al. (2018) focused on parental encouragement to diet in adolescence (i.e. direct communication only), the present study broadened the scope to explore how both retrospectively reported direct (e.g., encouragement to diet, comments about their weight) and indirect (e.g., parental dieting, parental comments about others weight) communication about weight and shape in childhood predicted mothers’ current weight/shape communication with their child. Although there is some debate on the validity and reliability of using retrospective reports of communication, this approach is common and research consistently shows a strong link between recalled childhood commentary and disordered eating in adulthood (Abraczinskas et al., 2012, Claydon et al., 2019, Heiman & Olenik-Shemesh, 2019, Kluck, 2010, Stice, 1998, Tan et al., 2016, Taniguchi, 2019, Vincent & McCabe, 2000, Wansink et al., 2017). Retrospective reports can provide insights into participants memories and help build a comprehensive picture of how past experiences may influence current behaviors (Abraczinskas et al., 2012, Claydon et al., 2019, Heiman & Olenik-Shemesh, 2019, Kluck, 2010, Stice, 1998, Tan et al., 2016, Taniguchi, 2019, Vincent & McCabe, 2000, Wansink et al., 2017).

Since overlap may exist between direct and indirect weight/shape communication, we also explored the interactive effects of childhood direct and indirect weight/shape communication. We hypothesized that women who endorsed direct weight/shape communication in childhood would be more likely to exhibit direct weight/shape communication with their child with a similar pattern emerging for indirect weight/shape communication. We did not have an a priori hypothesis for the interaction between childhood direct and indirect weight/shape communication given past studies have not explored these interactions.

## Methods

### Participants

Participants included 634 cisgender women who provided retrospective experiences of weight/shape communication from their parents and current self-reports of their weight/shape communication to their child. If the participant had more than one child in our target age range (age 6–11), a random number generator determined for which child the mother would answer questions. The age range of 6–11 years was chosen because it represents a critical developmental stage during which children begin to internalize messages about food, eating, and body image, often influenced by their parents (Davison & Birch, 2001; Lowes & Tiggemann, 2003). Parents also still play a significant role in shaping children’s eating behaviors compared to adolescence, where children begin to gain greater autonomy over their eating choices (Savage et al., 2007). This period is particularly important for examining conversations around food and eating habits, as children are just beginning to process and be shaped by parental influences (Birch & Fisher, 1998). Inclusion criteria included being a cisgender female with at least one child between the ages of 6–11 and a resident of the United States. Women were the targeted population due to the higher likelihood that mothers would be more directly involved in feeding their children (Berge, 2014; Daniels et al., 2020; Kotila et al., 2013), therefore more likely to discuss the topic of food and eating with their children (Dahill et al, 2021; Rodgers et al., 2009).

### Measures Direct Weight/Shape-Related Communication

Current direct communication was assessed with the following two items: “In the past month, how often have you or your spouse/partner made a comment to your child about their weight?” and “In the past month, how often have you or your spouse/partner encouraged your child to lose weight?” Responses were provided on a 4-point scale (“Never”, “Once per month”, “A few times per month”, “At least once per week”). Given these items were strongly correlated (*r* = 0.75, *p* = <0.001; see all item-level correlations in Supplementary Table 1) and had excellent internal consistency (α = 0.86), the average of these two items was used to assess current direct communication. Participants also provided retrospective reports of their parents’ direct communication during their childhood (i.e., “During your childhood, how often did your parents/guardians make a comment to you about your weight?”, “During your childhood, how often did your parents/guardians encourage you to lose weight?”). Responses were indicated on a 5-point scale (“Never”, “Less than once per month”, “Once per month”, “A few times per month”, “At least once per week”). These items correlated highly (*r* = 0.67, *p* = <0.001) and exhibited good internal consistency (α = 0.81), thus were averaged to assess childhood direct communication.

### Indirect Weight/Shape-Related Communication

Current indirect communication was assessed with the following items: “In the past six months, how often have you dieted?” given on a 5-point scale (“Never”, “Less than once per month”, “Once per month”, “A few times a month”, and “At least once a week”) and “In the past month, how often have you complained about your weight or how you look?”, given on a 4-point scale (“Never”, “Once per month”, “A few times per month”, “At least once per week”). Given these items were strongly correlated (*r* = 0.52, *p* < 0.001) and had adequate internal consistency (α = 0.66), the average of these two items was used to assess current indirect communication. Childhood indirect communication was assessed with the following items: “During your childhood, how often did your parents/guardians diet?”, and “During your childhood, how often did your parents/guardians complain about their weight or how they looked?” Responses were provided on a 4-point scale (“Never”, “Rarely”, “Often”, “Always”) and 5-point scale (“Never”, “Less than once a month”, “Once a month”, “A few times per month”, “At least once a week”), respectively. These items were strongly correlated (*r* = 0.56, *p* = <0.001) and had adequate internal consistency (α = 0.67), therefore were averaged to assess childhood indirect communication.

### Procedures

Participants were recruited to participate in an online study about the “impact of food availability on eating and feeding behaviors” using Amazon’s Mechanical Turk (MTurk). Approximately half the participants were recruited on the basis of having experienced food insecurity (i.e., a lack of reliable access affordable, nutritious food) in childhood which was assessed using three gateway items from the USDA Household Food Security Survey Module (United States Department of Agriculture [USDA], 2012) adapted to inquire about food security in childhood. The study was posted as a Human Intelligence Task (HIT) for workers to complete. HITs are the term used to describe the posted tasks that workers can do and involve many different activities such as answering questionnaires and surveys. The survey took an average of 28.49 min (SD = 22.31) to complete. Participants were compensated $5 for completion of the study. Participants provided informed consent online prior to completing the questionnaire. The University of Chicago’s Institutional Review Board approved all study procedures.

MTurk worker qualifications/requirements to participate included a HIT approval rate of >100 (i.e. more than 100 prior tasks (HITs) needed to have been completed and approved) and HITs approved >95% (i.e. more than 95% of their prior tasks (HITs) needed to have been approved and completed since registering with MTurk). These requirements were enacted to ensure quality responses and are consistent with other studies using MTurk samples (i.e., ≥90% approval rate, ≥100 approved HITs; Chmielewski & Kucker, 2019). In addition, six quality checks were included in the questionnaire. Participants were asked to select the one sentence that does not make semantic sense (e.g., “Planes yell on the dream”) from a set of four syntactically correct sentences (e.g., “Boats are sailing on the lake”). With the exception of one comparison [missing 0 quality checks (M = 26.47 min, SD = 11.92) and four quality checks (M = 17.84 min, SD = 10.29)], the average completion time of the survey did not differ based on the number of quality checks missed. Internal consistency of well-validated, reliable measures that included reverse scored items were explored across differing numbers of missed quality checks. Cronbach’s alpha decreased when participants who missed four or more quality checks were included. Therefore, individuals who incorrectly answered at least four of the six quality check questions were excluded from the study (*n* = 171). This resulted in our current analytic sample of 634 participants.

### Data Analysis

Multiple linear regression was used to explore whether childhood direct and indirect communication predicts parents’ current weight and shape communication towards their children. Six separate multilinear regressions were performed: (1) childhood direct communication predicting current direct communication, (2) childhood indirect communication predicting current direct communication, (3) childhood direct communication predicting current indirect communication, (4) childhood indirect communication predicting current indirect communication, (5) both childhood direct and childhood indirect communication predicting current direct communication, and (6) both childhood direct and childhood indirect communication predicting current indirect communication. The last two models (i.e., models 5 and 6) also included the interaction between childhood indirect and direct communication. The participants’ child’s BMI percentile, child’s gender, child’s age, family income, ethnicity, and race were included as covariates to ensure that the findings were not influenced by the child’s body size, gender, SES, or race/ethnicity. Age and gender-specific BMI percentiles were calculated from parent-reported height and weight using CDC growth charts (https://www.cdc.gov/healthyweight/xls/bmi-groupcalculator-us-062018-508.xlsm).

## Results

Participants had a mean age of 34.75 years (SD = 7.40, range 22–72), and the mean age of their children was 7.77 (SD = 1.72). Approximately, 45.2% of the participants’ children were reported female, 54.1% male, 0.2% transgender female, 0.2% non-binary, and 0.2% “other” gender identity. Participant household income represented a range of socioeconomic backgrounds with 18.0% reporting a household income <$30,000, 40.8% between $30,000 and $59,000, 23% between $60,000 and $89,999, and 18.3% ≥$90,000. The majority of participants identified as White (77.4%) followed by participants who identified as African American (18.3%), Asian (3.8%), American Indian/Alaskan Native (3.2%), Native Hawaiian/Pacific Islander (1.1%), and those who preferred not to provide their race (1.3%). Notably, participants were able to select more than one race; therefore, percentages do not add up to 100%. The majority of the sample identified as non-Hispanic (82.0%) with a small percentage not providing their ethnicity (2.4%).

**Table 1:** Descriptive statistics

	Mean (SD)	Range
Mother age	34.75 (7.40)	22–72
Child age	7.77 (1.72)	6–11
Child BMI Percentile	69.07 (31.81)	0–100
Household Income	6.24 (2.88)	1–13
Childhood Direct	0.94 (1.11)	0–4
Childhood Indirect	1.17 (1.01)	0–3.50
Direct	0.41 (0.71)	0–3
Indirect	1.19 (1.09)	0–3.50

Household income was assessed with the following: 1= Less than $10,000, 2= $10,000-$19,999, 3 = $20,000-$29,999, 4 = $30,000-$39,999, 5= $40,000-$49,999, 6= $50,000-$59,999, 7 = $60,000-$69,999, 8= $70,000-$79,999, 9 = $80,000-$89,999, 10= $90,000-$99,999, 11= $100,000-$149,999, 12 = $150,000-$199,999, 13= More than $200,000

*SD* standard deviation

A positive association was found between mothers reported childhood direct communication about weight/shape and their direct communication to their own child (*r* = 0.52, *p* < 0.001; see Table 1). Similarly, a positive association was found between mother’s experienced childhood indirect weight/shape communication and their current indirect communication towards their own child (*r* = 0.39, *p* < 0.001). Further, individuals who experience one communication style in childhood were also likely to experience the other as well, in that childhood direct communication was positively associated with childhood indirect communication (*r* = 0.49, *p* < 0.001). Similarly, current direct and indirect communication was significantly positively correlated (*r* = 0.35, *p* < 0.001). Regarding child BMI percentile and parent communication, a weak but statistically significant correlation between direct communication and child BMI percentile was found (r = 0.14, *p* < 0.001), whereas child BMI percentile was not significantly associated with indirect communication (r = 0.004, *p* = 0.94).

### Multi-Linear Regression

A series of regressions examined the associations between mother’s childhood and their current direct and indirect communication styles, adjusting for race, ethnicity, income, child age, child gender, and child BMI percentile (see Table 2). When mother’s childhood weight/shape communication is modeled independently, the mother’s current direct communication is predicted by their childhood direct (**Model 1**: B = 0.44, 95%CI [0.36,0.51]) and indirect (**Model 2**: B = 0.20, 95%CI [0.12,0.28]) communication. Similarly, mother’s current indirect communication is predicted by their childhood direct (**Model 3**: B = 0.31, 95%CI [0.23,0.40]) and indirect (**Model 4**: B = 0.37, 95%CI [0.29,0.45]) communication. However, when mother’s childhood direct and indirect communication are modeled simultaneously, only mother’s childhood direct communication (**Model 5**: B = 0.45, 95%CI [0.35,0.54]) predicts their current direct communication, and their childhood indirect communication is no longer a significant predictor. Interestingly, this is not the case for mother’s current indirect communication for which both mother’s childhood direct (**Model 6:** B = 0.23, 95%CI [0.13,0.33]) and indirect (**Model 6**: B = 0.28, 95%CI [0.19,0.37]) communication were significant predictors.

**Table 2:** Regression analysis with childhood direct and indirect weight/shape communication predicting current weight/shape communication

	R^2^(adj).	Predictor(s)	Outcome	B	SE	95% CI	t	p-value
Model 1	0.25	Childhood Direct	Current Direct	**0.44**	**0.04**	**(0.36, 0.51)**	**11.13**	**<0.001**
Model 2	0.11	Childhood Indirect	Current Direct	**0.20**	**0.04**	**(0.12, 0.28)**	**4.76**	**<0.001**
Model 3	0.10	Childhood Direct	Current Indirect	**0.31**	**0.04**	**(0.23, 0.40)**	**7.32**	**<0.001**
Model 4	0.14	Childhood Indirect	Current Indirect	**0.37**	**0.04**	**(0.29, 0.45)**	**8.92**	**<0.001**
Model 5	0.25	Childhood Direct		**0.45**	**0.05**	**(0.35, 0.54)**	**9.31**	**<0.001**
		Childhood Indirect		<0.01	0.04	(−0.08, 0.09)	0.09	0.93
		Childhood Direct * Childhood Indirect	Current Direct	−0.03	0.04	(−0.10, 0.05)	−0.64	0.53
Model 6	0.17	Childhood Direct		**0.23**	**0.05**	**(0.13, 0.33)**	**4.61**	**<0.001**
		Childhood Indirect		**0.28**	**0.05**	**(0.19, 0.37)**	**6.09**	**<0.001**
		Childhood Direct * Childhood Indirect	Current Indirect	**−0.11**	**0.04**	**(−0.19, −0.02)**	**−2.55**	**0.01**

Bold values denote statistically significant p-values of 0.01 or below

95% CI = 95% confidence interval Each model also included race, ethnicity, household income, child age, child gender, and child’s BMI percentile as covariates

*B* standardized beta, *SE* standardized error

**Fig. 1:**
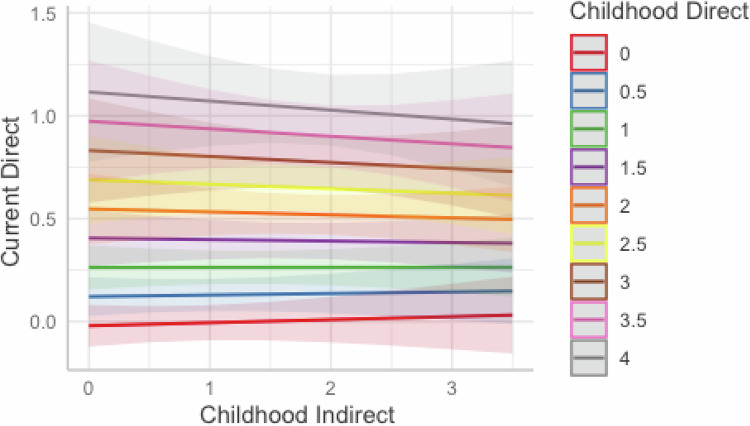
Interaction between childhood direct and indirect weight/shape communication in predicting current direct weight/shape communication. The numbers in the colored boxes represent the level of direct weight/shape communication the mother reported during childhood with 0 indicating no/low amounts of childhood direct weight/shape communication and 4 indicating very high amounts of mother’s childhood direct weight/shape communication. The interaction between mother’s childhood direct and indirect weight/shape communication in predicting their current direct communication was non-significant. There is a positive association between mother’s childhood direct weight/shape communication and current direct weight/shape communication (as seen by higher levels of childhood direct communication (different colored lines) being associated with higher levels of current direct weight/shape communication (y-axis); however, this relationship remains unchanged across levels of childhood indirect weight/shape communication (x-axis). Race, ethnicity, household income, child age, child gender, and child’s BMI percentile were included as covariates in the model

**Fig. 2:**
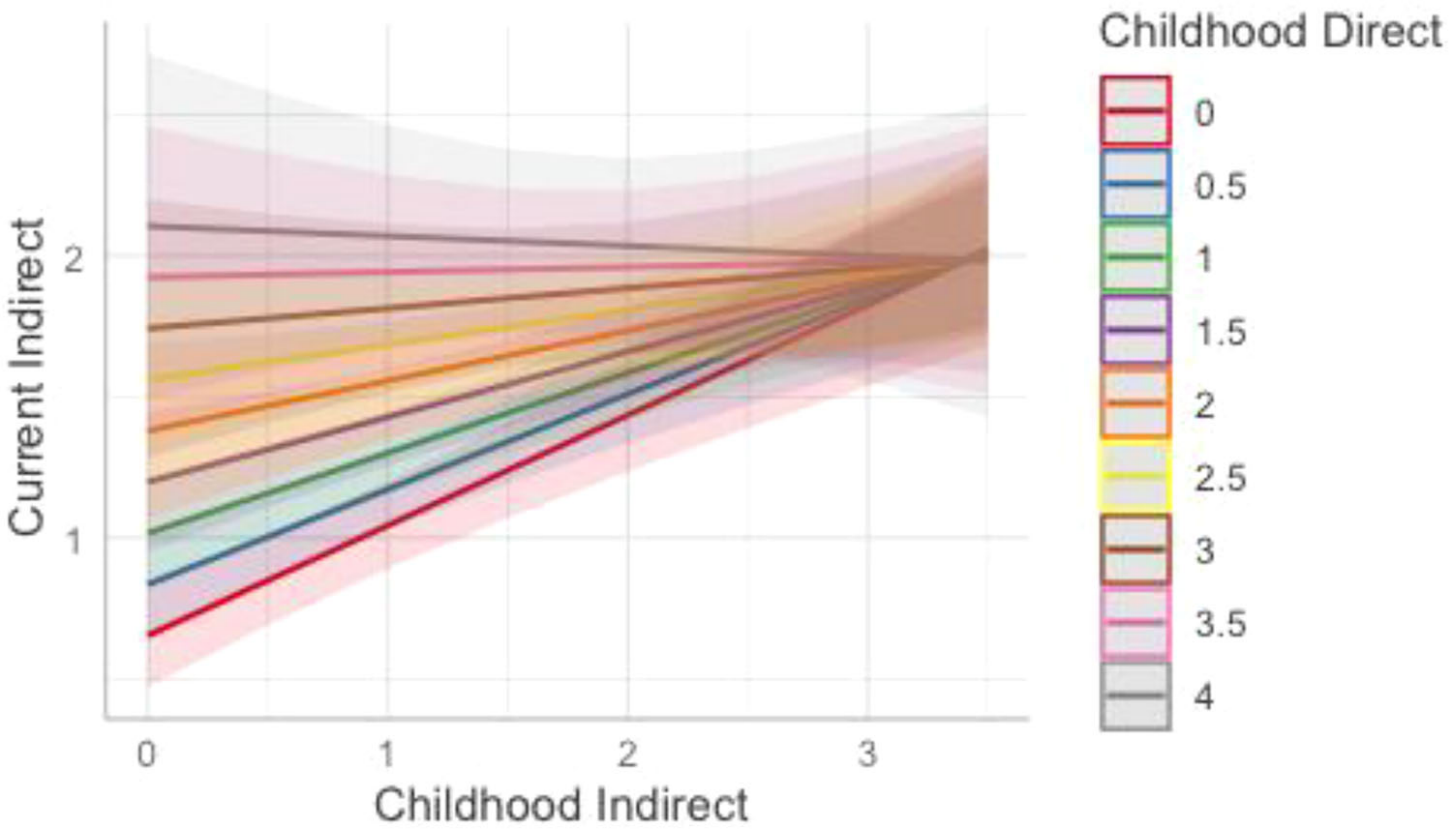
Interaction between childhood direct and indirect weight/shape communication in predicting current indirect weight/shape communication. The numbers in the colored boxes represent the level of direct weight/shape communication the mother reported during childhood with 0 indicating no/low amounts of childhood direct weight/shape communication and 4 indicating very high amounts of mother’s childhood direct weight/shape communication. There was a significant interaction between mother’s childhood direct and indirect weight/shape communication in predicting current indirect communication. At lower levels of childhood direct communication (represented by the red, blue, green lines), childhood indirect communication (along the x-axis) is more predictive of current indirect communication (y-axis), whereas at higher levels of childhood direct weight/shape communication, childhood indirect communication is less predictive of current indirect communication (as seen with the nearly flat lines). Race, ethnicity, household income, child age, child gender, and child’s BMI percentile were included as covariate in the model

As noted, Models 5 and 6 also included a childhood direct-childhood indirect interaction term as a predictor of current direct (**Model 5**) and current indirect (**Model 6**) communication. As seen in Fig. 1, this interaction was not significant when predicting current direct communication (B = −0.03, 95%CI [−0.10,0.05]). Experiencing indirect communication about weight/shape in childhood does not change the relationship between childhood direct communication and current direct communication. However, the interaction between childhood direct and indirect communication significantly predicted current indirect weight/shape communication (B = −0.11, 95%CI [−0.19,−0.02]). As seen in Fig. 2, for mothers who experienced high levels of direct communication about weight/shape in their childhood, childhood indirect communication appears to be less influential in predicting current indirect communication. For mothers who experienced low levels of direct communication in childhood, childhood indirect communication significantly influenced their indirect communication to their children in adulthood.

## Discussion

Parent weight/shape-based communication has been consistently associated with offspring eating pathology (Abraczinskas et al., 2012; Arroyo et al., 2017; Berge et al., 2018; Berge et al., 2024; Claydon et al., 2019; Dahill et al., 2021; Martini et al., 2020; Norton et al., 2022; Rodgers et al., 2009; Rodgers & Chabrol, 2009; Savage et al., 2007; Stice, 1998); however, fewer studies have explored what factors drive this type of communication. Using a well-powered independent sample, the present study found that parental communication about weight/shape in childhood is associated with one’s own weight/shape communication towards their children in adulthood. Consistent with the studies by Berge et al. (2018), (2024), women who experienced direct comments from their parents about their weight/shape during their childhood are more likely to directly comment on their own child’s weight, as well as model the importance of weight/shape through their own dieting and comments about their body as adults. Further, women who as children observed their parents commenting on their own weight and/or engaging in dieting were more likely as mothers to comment on their child’s weight (when modeled independently from childhood direct weight/shape communication), their own weight, and/or engage in dieting.

To our knowledge, this study was the first to explore the interactions between types of weight/shape communication experienced in childhood and their relationship to current weight/shape parental communication in mothers. Interestingly, women who observed their parents dieting or commenting about their own body in childhood (i.e., indirect communication) did not significantly predict current commentary about their own offspring’s weight when woman’s childhood weight/shape commentary (i.e., direct communication) was included in the model. However, women who observed their parents dieting or commenting about their own body in childhood was predictive of similar behavior in adulthood when woman’s childhood weight/shape commentary was minimal. Taken together, compared to indirect weight/shape communication, experiencing direct comments about weight or encouragement to diet in childhood appears to be more consistently predictive of future weight/shape communication for women. However, in the absence of direct communication, parental modeling of the importance of weight/shape through dieting or commenting about their body is associated with similar behaviors for the offspring in adulthood. All associations remained even when controlling for the child’s BMI percentile, suggesting that these associations are not necessarily driven by the child’s body size.

Prior research highlights the importance of exploring potential environmental risk factors for disordered eating that may influence children and early adolescent offspring. Indeed, while parents may influence their offspring’s level of eating pathology via genetic main effects (e.g., parents passing along genetic vulnerability for the development of disordered eating), shared environmental effects (e.g., parents modeling the importance of weight/shape within the home), or gene-environment interplay (e.g., offspring inheriting both genetic vulnerability for disordered eating and being reared in an environment shaped by those genes), past developmental twin studies have highlighted the importance of environmental factors during this developmental period with heritability estimates for disordered eating largely 0% in early adolescence, pre-puberty and approximately 50% during late adolescence, mid-to-late puberty and into adulthood (Klump et al., 2003; 2007; 2009; 2017; O’Connor et al, 2020; Wade et al., 2012). In fact, recent studies have further highlighted a lack of genetic influence in childhood/pre-puberty by demonstrating both a lack of genetic main effects and a lack of evidence of gene-environment interplay via passive gene-environment correlation (O’Connor et al., 2019; O’Connor et al., 2022). Thus, future work is needed to further elucidate specific environmental factors important for disordered eating development during childhood and early adolescence. Interestingly, the findings from this study suggest that the way parents communicate about weight/shape during childhood may, not only act as an environmental stressor but have lasting and intergenerational ramifications on weight/shape communication approaches that are known to be associated with elevated disordered eating.

Findings from the present study highlight a need to increase psychoeducation for parents on the possible effects of weight-based commentary during childhood and adolescence. Indeed, regardless of the intention behind a weight-based comment, these comments may increase risk for the development of disordered eating behaviors in their offspring (Abraczinskas et al, 2012; Arroyo et al., 2017; Berge et al., 2018; Claydon et al., 2019; Martini et al., 2020; Rodgers et al., 2009; Rodgers & Chabrol, 2009; Savage, et al., 2007; Stice, 1998), and further perpetuate messages about the importance of low body weight/thin body shapes to future generations via direct and indirect communication, as suggested in the present study.

While our study has many strengths (e.g., well-powered sample, novel exploration of communication style interactions), it is not without limitations. First, our data was collected using Amazon’s Mechanical Turk (MTurk) which has been thought to exhibit low levels of reliability, potentially resulting from insufficient attention while responding (Goodman et al., 2012). Notably, however, our study required MTurk worker qualifications that aimed to recruit individuals who consistently and reliably engaged on the platform. Further, we embedded multiple attention checks to ensure participants were adequately attending to the survey. The present study also relied on a single informant’s current self-report and retrospective self-report experiences. Retrospective-report and self-report data may not reflect the objective experience of the individual and thus, may be a bias representation. However, retrospective reports highlight the informant’s perception of childhood messages, which still provide valuable insights as perceived experiences can be just as important as objective ones (Danese & Widom, 2020). Additionally, our data was cross-sectional, thereby limiting the predictive validity of the findings. Future studies would benefit from using longitudinal approaches with multiple informants. Furthermore, the key constructs (i.e., current and retrospective reports of direct and indirect communication) were assessed using only two items each which could weaken the study’s ability to capture the full breadth and complexity of these types of these communication styles. Child’s BMI was also collected by parent’s self-report of their child’s height and weight, which could lead to potential reporting inaccuracies. Finally, the biggest limitation of the study involved the questions used to assess current direct communication. These questions ask, “Do you or your partner….” and thus, it is possible that the participant is reporting on their spouse or partner’s behavior when reporting on current direct communication with their child. Interestingly still, the mother’s childhood weight/shape communication was predictive of her (or her partner’s) current direct communication. Additionally, research suggests that mothers communicate more with their children than fathers in general (Benedikt et al., 1998; Leaper et al., 1998) as well as engage in more frequent conversations about eating, weight, and shape (Dahill et al, 2021; Rodgers et al., 2009). Therefore, most participants are likely reporting on themselves and not their partner. Research suggests a reasoning behind this phenomenon could be due traditional caregiving roles (Moura & Philippe, 2023). For example, mothers are frequently seen as the “primary caregivers” in the family, which includes overseeing food preparation and establishing family dietary habits. This caregiving role extends to discussions about body image, dieting behaviors, and healthy eating, where mothers tend to model or promote healthy behaviors more actively than fathers (Dahill et al., 2021; Moura & Philippe, 2023). In contrast, fathers often engage less in these conversations, possibly due to societal perceptions of masculinity that distance men from caregiving activities like food management (Moura & Philippe, 2023). Studies indicate that some fathers may even perceive less responsibility for feeding children and may defer to mothers in these areas (Daniels et al., 2020; Moura & Philippe, 2023). However, the wording of the question still does pose as a limitation potentially breaking the intergenerational link. Future studies should strictly define whether the participant is answering based on their own or their partner’s direct communication.

Parental influence has long been purported as an important factor in offspring disordered eating development (Dahill et al., 2021, Norton et al., 2022). Specifically, parental communication through direct commentary or indirect modeling of disordered eating have been linked to higher disordered eating in the offspring (Arroyo et al., 2017; Berge et al., 2018; Claydon et al., 2019; Gillison et al., 2016; Lydecker et al., 2018; Rodgers et al., 2009; Rodgers & Chabrol, 2009; Tan et al., 2016). Our study demonstrates that the direct and indirect communication that parents receive in childhood may influence their communication about weight/shape to their children. Notably, experiencing direct commentary in childhood is a strong influence on both later indirect and direct communication. It is also important to note that childhood indirect communication about weight/shape significantly predicts current indirect communication demonstrating that children may still absorb indirect messages even when direct communication is not present. Greater attention to the importance of families breaking the intergenerational cycle of dieting and weight commentary may protect future generations from unhealthy weight control behaviors and beliefs.

## Supplementary information

Supplementary Table 1

## Data Availability

Data can be made available upon request.
