# Supplementary material for: Parent to Child Intergenerational Transmission of Direct and Indirect Weight and Shape Communication
Source: J Child Fam Stud. 2025 Aug 14;34(8):2070–80. doi: 10.1007/s10826-025-03078-z (PMC12394254; doi:10.1007/s10826-025-03078-z)
Supplement: Supplementary file 1 — Supplementary Table 1 [file 10826_2025_3078_MOESM1_ESM.docx]

Supplemental Table 1. Item level correlations

|  | Childhood Parent Weight Comment | Childhood Parent Encouragement to Diet | Childhood Parent Weight Complaints | Childhood Parent Dieting | Current Comments about Child’s Weight | Current Encouragement to Diet | Current Weight Complaints | Current Dieting |
| --- | --- | --- | --- | --- | --- | --- | --- | --- |
| Childhood Parent Weight Comment | 1.00 | - | - | - | - | - | - | - |
| Childhood Parent Encouragement to Diet | .67*** | 1.00 | - | - | - | - | - | - |
| Childhood Parent Weight Complaints | .46*** | .39*** | 1.00 | - | - | - | - | - |
| Childhood Parent Dieting | .34*** | .39*** | .56*** | 1.00 | - | - | - | - |
| Current Comments on Child's Weight | .42*** | .49*** | .22*** | .27*** | 1.00 | - | - | - |
| Current Encouragement to Diet | .38*** | .51*** | .20*** | .26*** | .75*** | 1.00 | - | - |
| Current Weight Complaints | .34*** | .31*** | .34*** | .32*** | .41*** | .36*** | 1.00 | - |
| Current Dieting | .23*** | .21*** | .29*** | .29*** | .22*** | .22*** | .52*** | 1.00 |

*Note*. Childhood parent weight comment = “During your childhood, how often did your parents/guardians make a comment to you about your weight?”; Childhood parent encouragement to diet = “During your childhood, how often did your parents/guardians encourage you to lose weight?”; Childhood parent weight complaints = “During your childhood, how often did your parents/guardians complain about their weight or how they looked?”; Childhood parent dieting = “During your childhood, how often did your parents/guardians diet?”; Current comments about child’s weight = “"In the past month, how often have you or your spouse/partner made a comment to your child about their weight?"; Current encouragement to diet = “In the past month, how often have you or your spouse/partner encouraged your child to lose weight?”; Current weight complaints = “In the past month, how often have you complained about your weight or how you look?”; and Current dieting = “In the past six months, how often have you dieted?”

*p<.05, **p<.01, ***p<.001
